# The future of viral hepatitis testing: innovations in testing technologies and approaches

**DOI:** 10.1186/s12879-017-2775-0

**Published:** 2017-11-01

**Authors:** Rosanna W. Peeling, Debrah I. Boeras, Francesco Marinucci, Philippa Easterbrook

**Affiliations:** 1 Depart of Clinical Research and International Diagnostics Centre, London School of Hygiene and Tropical Medicine, Keppel Street, London, WC1E 7HT UK; 2 Foundation for Innovative New Diagnostics, Geneva, Switzerland; 3 Global Hepatitis Programme, HIV Department, World Health Organization, Geneva, Switzerland

**Keywords:** Hepatitis B virus, Hepatitis C virus, Point-of-care, Diagnostic test, Low resource settings, WHO, Innovations

## Abstract

A large burden of undiagnosed hepatitis virus cases remains globally. Despite the 257 million people living with chronic hepatitis B virus infection, and 71 million with chronic viraemic HCV infection, most people with hepatitis remain unaware of their infection. Advances in rapid detection technology have created new opportunities for enhancing access to testing and care, as well as monitoring of treatment. This article examines a range of other technological innovations that can be leveraged to provide more affordable and simplified approaches to testing for HBV and HCV infection and monitoring of treatment response. These include improved access to testing through alternative sampling methods (use of dried blood spots, oral fluids, self-testing) and combination rapid diagnostic tests for detection of HIV, HBV and HCV infection; more affordable options for confirmation of virological infection (HBV DNA and HCV RNA) such as point-of-care molecular assays, HCV core antigen and multi-disease polyvalent molecular platforms that make use of existing centralised laboratory based or decentralised TB and HIV instrumentation for viral hepatitis testing; and finally health system improvements such as integration of laboratory services for procurement and sample transportation and enhanced data connectivity to support quality assurance and supply chain management.

## Background

It is estimated that in 2015, 257 million people were living with chronic hepatitis B virus (HBV) infection and 71 million with chronic viraemic hepatitis C (HCV) infection [1, 2]. The African and Western Pacific regions account for 68% of those infected with HBV, while the European and Eastern Mediterranean regions are more affected by HCV. Untreated HBV and HCV infection can lead to life-threatening complications of cirrhosis and hepatocellular carcinoma accounting for 720,000 and 470, 000 annual deaths, respectively [1, 3]. Mortality from viral hepatitis has also increased 22% since 2000 and unless diagnosed and treated, the number of deaths due to viral hepatitis will continue to increase [4]. Yet, the majority of these infections remain undiagnosed globally. In 2015, only 9% of HBV infected persons and 20% of HCV were tested and aware of their diagnosis [1]. The 2016 World Health Organization (WHO) global health sector strategy on viral hepatitis has now set ambitious targets to eliminate viral hepatitis as a public health threat by 2030 (defined as a 65% reduction in mortality and a 90% reduction in incidence compared with the 2015 baseline) [5].

In this article we review existing technologies (serological and virological) for hepatitis testing and recommendations for testing from the 2017 WHO Guidelines on Hepatitis B and C testing [6]. We identify remaining challenges and explore technological advances and innovations that can help countries address these challenges and reach the WHO targets. These include improved access to testing through alternative sampling methods (use of dried blood spots, oral fluids, self-testing); more affordable options for confirmation of virological infection (HBV DNA and HCV RNA) that include point-of-care molecular assays, HCV core antigen and multi-disease (HIV/TB/HCV/HBV) polyvalent molecular platforms that make use of existing centralised laboratory based or decentralised instrumentation for viral hepatitis testing; and finally health system improvements such as integration of laboratory services for procurement and sample transportation and enhanced data connectivity to support quality assurance and supply chain management.

## Current testing technologies

### Serological testing

Serological assays are typically used as the first line of the testing strategy to screen for exposure to a virus and detect the host immune response (antibodies to HCV) or a viral antigen (HBsAg, HCVcAg). Hepatitis serological assays are based on the immunoassay principle, and are available in the form of rapid diagnostic tests (RDTs) or laboratory-based enzyme immunoassays (EIAs), chemoluminescence immunoassays (CLIAs) and electro-chemoluminescence immunoassays (ECLs). These latter assays offer excellent precision and reliability, high-speed throughput, random access, and the technical simplicity of full automation for high-volume clinical laboratories. Rapid diagnostic tests (RDTs) provide improved access to testing as they are simple, low cost, and can use serum and plasma, as well as capillary whole blood collected with a finger prick. They can also be performed by trained lay providers or health-care workers in resource-limited settings without the need for specialized equipment or venepuncture. Current costs for EIA assays range from 1 to 9 USD per test and RDTs range from 0.5 – 7USD.

### Virological testing

Nucleic acid testing (NAT) technologies are highly accurate in detecting the presence of viral nucleic acid (DNA or RNA), and are used to confirm either the presence or absence (qualitative) or level (quantitative) of viraemia to guide treatment decisions and monitor treatment response, and confirm either virological cure (HCV) or effective suppression (HBV). Hepatitis NAT assays are traditionally performed as laboratory based assays requiring highly trained staff and their current costs which range from 30 to 120 USD are unaffordable for majority of patients in low-resource settings.

## Recommended algorithms for hepatitis testing in 2017 WHO testing guidelines [6]

Overall, the guidelines recommend the use of a single quality-assured serological in vitro diagnostic test (i.e.either a laboratory-based immunoassay [EIA or chemiluminiscence immunoassay] or RDT) to detect HBsAg and HCV antibody that meet minimum performance standards [6]. The use of HBV DNA NAT following a reactive HBsAg serological test result has been recommended to help further guide treatment and monitoring where available [7]. Following a reactive HCV antibody serological test result, the 2017 WHO Guidelines Development Group made a strong recommendation for the use of either qualitative or quantitative NAT for diagnosis of HCV infection, with a qualitative assay being considered sufficient now in the era of highly effective direct acting antiviral regimens [8]. A systematic review was conducted to determine the accuracy of qualitative versus quantitative NAT methods for HCV RNA for detection (and/or) quantification to confirm viraemic HCV infection. The lower limit of detection was in the 10 to 15 IU/mL range for most qualitative assays, and 600–1100 IU/mL for quantitative assays. Table 1 summarises the included studies - the sensitivity of HCV viral quantitative assays was 87–100% compared to qualitative assays and most assays were able to detect all genotypes [9–12]. Since the review, six new quantitative molecular technology platforms have become available with linear ranges of quantitation between 12 to 10^8^ IU/mL and lower limits of detection of 15 IU/mL, which is comparable to qualitative assays. However, quantitative assays still provide a reproducible method to detect and quantify HCV RNA in plasma or serum, especially in the context of research studies. It was also observed that most qualitative or quantitative NAT assays as well as core Ag will capture the majority of viraemic infections, since 95% of those with chronic infection have a viral load > 10,000 IU/mL [13].

**Table 1:** Diagnostic performance of qualitative vs quantitative HCV RNA NAT in five studies included in 2017 systematic review

Author, year and country	Sample type & number	Study population	Diagnostic test (Quantitative)	Reference test (Qualitative)	Sensitivity	Specificity
Lee et al., 2000, USA [9]	Serum *N* = 51	Patients at risk of HCV infection	Roche AMPLICOR HCV test, version 2.0	Roche COBAS AMPLICOR TM HCV Test v2.0	100% at 50 IU/mL; 91% at 25 IU/mL*	
Yu et al., 2000, Taiwan [10]	Serum *N* = 215	Patients with chronic HCV	Bayer bDNA-2 Quantiplex HCV test	Roche COBAS AMPLICOR TM HCV Test v2.0	95%	–
Sabato et al., 2007, USA [11]	Plasma *N* = 76	Patients with HCV infection	*Bayer* Versant HCV genotype assay	Roche COBAS AMPLICOR TM HCV Test v1.0	100%	100%
Vermehren et al., 2008, Germany [12]	Serum *N* = 65	Patients with HCV	Bayer Versant HCV genotype assay	Abbott Real-Time Assay	87%	–

*CAP/CTM Roche Cobas Ampliprep/ Cobas Taqman HCV assay, *IU* International Units

In settings where NAT for confirmation of HCV viraemic infection is not available, the WHO guidelines recommended that HCV core antigen with comparable clinical sensitivity to NAT technologies could be considered as a potentially more affordable alternative [6]. This was supported by a systematic review of the diagnostic accuracy of seven commercial HCVcAg assays compared to NAT assays. Certain commercial HCVcAg assays had a high sensitivity (up to 93.4%) and a high specificity (> 98%) [13].

## Technological advances and innovations to improve access to testing

Understanding the major reasons for the current low rate of hepatitis testing in resource limited settings is critical to inform optimal positioning of new technologies to expand access to testing and treatment [14]. In addition to the current limited number of clinics and settings offering viral hepatitis testing and low demand from general population due to the lack of awareness on viral hepatitis, the costs of assays to confirm virological infection remain high and unaffordable for many hepatitis control programmes in low and middle income countries [15]. In recent years, diagnostic innovations in many areas of health have been driven by well-funded high priority diseases such as HIV and tuberculosis, global health emergencies and epidemic preparedness and information technology and data connectivity [16]. Examples of these innovations include HIV self-testing, mobile health applications, integration and co-location of HIV/AIDS tuberculosis and drug treatment services [17–20]. Table 2 summarizes a range of strategies and technological innovations that can be leveraged to improve access to HBV and HCV testing and treatment monitoring.

**Table 2:** Summary of innovative technologies to provide more affordable and simplified HBV and HCV testing and treatment monitoring

Area of Innovation	Technology or strategy	Primary testing target	Potential future testing target	Potential impact
1. Simplification of algorithms	• Elimination of need for genotyping with access to pan-genotypic DAAs, and only a single time point (SVR12) for assessment of cure	HCV		Reduce costs; Improve uptake
2. Sampling approaches	• Dried blood spots (DBS) • Oral fluid	• HCV • HCV		Increase access and coverage, reaching key and target populations
3. Innovative testing approaches	• Self-testing • Combo integrated multi-disease tests • Integrated multidisease testing platforms (centralised and decentralised)	• HCV • HCV • HCV	• HBV • HBV	Increase awareness, reduce stigma; Maximise programme synergies and reduce costs. Improve access to testing,
4. New technologies	• Point-of-care (POC) NAT • HCV core antigen test (as laboratory based assay and in future as POC)	• HCV • HCV	• HBV	Increase access to confirmation of viraemia
5. Health system improvements	• Integrated testing and service decentralization • Data connectivity • Sample chain and supply management	• HCV	• HBV	Improve timely receipt of results and linkage to care; improve supply chain management; Optimize use of current infrastructure, sample workflow

### Simplification of testing algorithms to improve uptake and reduce costs

The WHO Guidelines on Hepatitis B and C Testing (2017) provide the basis and rationale for simplification of testing algorithms to increase affordability and so in turn uptake of testing [6]. First, the availability now of pan-genotypic DAA drugs eliminates the need for costly genotyping which is largely unavailable in resource limited settings. Second, the high rates of cure with DAA regimens means that only a single test of cure is required and no on-treatment monitoring. Table 3 summarises this evolving simplification in testing strategies for HCV infection, and associated potential cost reductions. In the future, considerations for one-step diagnostic approaches using either HCV core antigen or HCV RNA assays in high-prevalence populations (ie. > 70% seroprevalence), such as persons who inject drugs or in prisons may also offer further simplification, reducing loss-to-follow-up and enabling same-day treatment initiation.

**Table 3:** Simplifying testing algorithm and lowering the cost of monitoring treatment for HCV in the future

	Screening for exposure	Confirm viremia	Guide treatment	Treatment baseline/monitoring	Post-treatment	Cost of testing
Current Testing scenarios	EIA/RDT	RNA qual	RNA quant Genotyping	Toxicity RNA quant	RNA qual	*~220–1100 USD*
Future Testing scenarios	EIA/RDT/Oral/DBS	RNA qual/quant	NA	RNA qual/quant	RNA qual/quant	*~15–75 USD*

### New sampling approaches

#### Dried blood spots for serology and virology to promote testing uptake and linkage to care

Dried blood spot (DBS) specimens have been used successfully to expand access to HIV testing due to the low cost, relative ease of specimen collection with avoidance of venepuncture, easier handling that does not require high skill, transportation with lower biohazard risk, and easier storage options [21]. The 2017 WHO Guidelines Development Group recognized the benefits of DBS and in particular their potential to facilitate greater access to testing and recommended considering DBS for both serological and NAT HBV and HCV technologies [6]. Four systematic reviews and meta-analyses on the diagnostic accuracy and impact of using DBS specimens for hepatitis B and C serological and NAT testing showed generally high diagnostic accuracy and precision of DBS specimens for both serology testing and NAT [22, 23]. DBS specimens were generally stable over time and maintained good accuracy in conditions with higher temperatures or humidity.

Current research priorities include the validation of commercial assays, both serological and molecular, using DBS, and to assess their performance in different populations and settings. The availability of DBS, both for serology testing and NAT, could allow a one-sampling strategy where the same DBS card is initially used with serology for screening and then reflex to NAT in those samples that are antibody positive. This strategy, if properly implemented, might result in major cost savings and expansion of testing. The Foundation for Innovative New Diagnostics (FIND), through a grant from UNITAID, is supporting manufacturers with the evaluation and optimization of both serology and molecular assays using DBS specimens. These studies, conducted initially in a reference laboratory and then in the field, aim to accelerate the submission of DBS protocols for regulatory approval (i.e. European CE IVD or WHO PQ) [24].

#### Testing using oral fluids

RDTs increase testing opportunities as their simplicity and flexibility allows use of multiple specimen types (e.g. venous, fingerstick blood, and oral fluid). In the field of HIV, the use of oral HIV RDTs for community based or home self-testing has increased access to testing for those who are marginalised or reluctant to visit clinics for testing due to stigma, and has been particularly successful among men who have sex with men [25, 26]. It would be anticipated that use of oral fluids for HBsAg and HCV testing would also increase access to testing in hard to reach populations including those in rural settings.

For viral hepatitis, use of oral fluids is currently only available with one quality approved HCV antibody RDT. Lee and colleagues evaluated the performance of the OraQuick® HCV Rapid Antibody Test in at-risk and symptomatic patients comparing the diagnostic performance to venous blood, fingerstick capillary blood, serum and plasma [9]. The overall sensitivities and specificities were almost identical (sensitivity, 99.7–99.9% and specificity, 99.6–99.9%) for all five specimen types. The observed sensitivity of the OraQuick test was slightly lower for oral fluid at 98.1% though the upper CI (99.0%) was equal to the lower CI for venous blood and fingerstick blood. Most of the study subjects with false negative OraQuick test results had serological and virological results consistent with resolved infection. Sensitivity for anti-HCV in early seroconversion was virtually identical between the HCV rapid test and EIA. The OraQuick® HCV Rapid Antibody Test demonstrated clinical performance that was equivalent to current laboratory-based EIAs. Major limitations to expanded use of oral testing are the current high costs of the OraQuick assay [27].

### Innovative testing approaches

#### Self-testing

Self-testing is currently an area of active research and implementation in HIV to improve access and uptake among specific populations, such as men who have sex with men or persons who inject drugs [28]. Multiple studies now show that HIV self–testing (HIVST) is acceptable among different high-risk populations who may not otherwise have access to testing. Mokgatle and colleagues assessed the acceptability of HIVST among students in two provinces in South Africa [29]. Overall, 87.1% and 75.6% of participants were willing to confirm HIVST results at a local health facility. In Zimbabwe and Malawi, Indravudh and colleagues found that HIVST was highly acceptable to young people largely because it empowers them to choose location and timing of the test and control disclosure of results [30]. High acceptability of self-testing for HIV status using an oral fluid-based RDT was observed among pregnant women in rural India [31]. In a survey among MSM in the UK, the primary perceived benefit of HIVST was privacy and confidentiality, with convenience cited as another benefit among those who had difficulty accessing services [32]. Based on country commitments to date, at least 4 million HIVST kits are expected to be procured by donor-funded programmes from July 2017 to the end of 2018 [33]. It remains unclear whether self-testing might play a similar role in promoting uptake of viral hepatitis testing among high risk populations. A key difference between HCV and HIV testing, is that the former still requires a test of viraemia to confirm need for treatment, while for HIV, the serological antibody test alone is sufficient.

Demonstration projects are therefore required to examine the acceptability and utility of HCV self-testing (using either capillary or oral fluid) in high risk populations or in remote settings, but will first require development of validated self-testing protocols for use with the OraQuick assay. These projects might also examine the strategies to enhance linkage to HCV RNA following a positive self-test, particularly for high risk stigmatised groups with poor access to health services (e.g. MSM, PWIDs) [34, 35]. If operational research proves the utility of HCV ST as approach to improve uptake of HCV screening this might prompt the development of a broader range of products, with associated cost reductions.

#### Combination integrated multi-disease tests

As HIV-infected persons continue to live longer due to increased uptake of anti-retroviral therapies, liver disease has emerged as a leading cause of morbidity and mortality in persons co-infected with HBV or HCV. Two recent systematic reviews and meta-analyses highlight the overlapping epidemics of HIV-HBV and HIV-HCV co-infection [36, 37]. As twin epidemics in key populations, combination integrated multi-disease blood or oral based assays that allow for integrated testing of HIV, HBV and HCV using a single sample would improve the efficiency of screening programmes. While not yet fully validated, preliminary results appear promising. Fisher and colleagues examined the sensitivity and specificity of different multiplex POC tests including MedMira rapid antibody test (whole blood; HIV/ HCV, HBV/HIV/HCV), Chembio (whole blood, oral specimens; HCV, HIV/HCV, HIV/HCV/Syphilis), OraSure (whole blood; HCV) [37]. Table 4 summarises the key findings which demonstrated that the OraSure and Chembio blood tests (including those multiplexed with HIV and syphilis) have good performance characteristics.

**Table 4:** Performance of HIV/HBV/HCV multiplex POC tests [38]

Company	Specimen	Sensitivity (95% CI)	Specificity (95% CI)
OraSure	HCV Antibody Test	92.7% (88.8%–96.5%)	99.8% (99.4%–100%)
Chembio	HCV Screen	92.1% (87.7%–96.4%)	99.2% (98.6%–99.9%)
	HIV-HCV Assay	91.5% (87.2%–95.7%)	99.4% (98.8%–99.9%)
	HIV-HCV-Syphilis	92.3% (88.4%–96.2%)	99.3% (98.8%–99.9%)
MedMira	HIV/HCV	79.1% (72.6%–85.5%)	100%
	HIV/HCV/HBV	81.5% (75.2%–87.8%)	100%

A further innovation in HCV serology is a combination RDT for the simultaneous detection of anti-HCV and core antigen, allowing for early detection of recent HCV infection. The HCV core Ag assay has shown to be as effective as NAT in detecting HCV infection, and about 6–7 weeks earlier than third generation anti-HCV assays [38, 39]. The fourth generation Ab-Ag assays are based on the concurrent detection of core Ag and anti-HCV and become positive as early as the HCV RNA assay. This can improve diagnosis of acute hepatitis C infection, especially in high-risk group populations such as PWID [40, 41]. In HIV/HCV co-infected patients the HCV seroconversion if often delayed with the consequence that a conventional anti-HCV screening test fails to react. The development of new RDTs combining the simultaneous detection of anti-HCV and core antigen may also improve detection of hepatitis C infections among HIV infected persons.

#### Integrated multidisease testing platforms (centralised and decentralised)

In addition to the use of multidisease combo RDTs, several laboratory molecular platforms allow for use of several disease-specific assays on the same platform. This applies both to platforms designed for high-throughput central laboratories as well as “near patient” technologies deployed closer to patients in lower level health facilities. The legacy of vertical disease programmes is that many laboratories have polyvalent platforms used only by the disease programme that procured it. In addition to the frequent underutilization of the platform, this has also resulted in parallel systems operating for different diseases in the same laboratory in terms of procurement, sample workflow, infrastructure and even staff salaries.

The integration of multiple assays on the same platforms has numerous benefits. The same sample referral system can be used across diseases and improve efficiencies in sample workflow. End users can be trained on multi-disease testing, improving their expertise and experience in strengthening the quality of testing across diseases. Inventory management, procurement and supply chain will improve and eliminate redundancies. Lastly, the use of multiple assays on the same platform will increase volume of tests from a single manufacturer and may be used in price negotiations to achieve bundled prices across diseases.

Sharing the same laboratory platform/instrument across different diseases makes strategic sense, but will require substantial re-organisation of laboratory processes, human resources and cost centres. In many LMICs, this will require active collaboration between different national disease programmes and public health laboratory services. A recent WHO technical update provided a strategic overview of key implementation considerations for diagnostic integration [42]. This document highlights that a country-led and country-coordinated collaborative process is essential to achieve integration, and that this should be a priority for both those countries that already have operational multidisease testing devices, as well as those considering their introduction. One key aspect in the selection of polyvalent platforms and selecting sites for deployment is clear mapping of all health facilities based on the catchment area, target patient populations across diseases and anticipated testing volumes. Donors and manufacturers also need to recognise and support the added value of platform sharing towards health system strengthening as well as more predictable reagent forecasting. All the platforms listed in Table 3 offer a broad menu of molecular assays covering multiple infectious diseases such as HIV, tuberculosis, viral hepatitis, STIs and respiratory tract infections. Pilot demonstration projects on assay integration into polyvalent platforms are needed to assess the capacity of the system (i.e. logistics and human resources) to achieve sharing across disease programmes. These projects can also help define placement criteria and inform implementation at country level.

### New technologies

#### Point-of-care (POC) molecular technologies (quantitative and qualitative)

NAT assays can now be performed on POC devices outside of laboratory settings. Point-of-care testing or near patient testing is defined as medical diagnostic testing at or near the point of care—that is, at the time and place of patient care. The delivery of health care at the most peripheral levels of the health system has played a critical role in antiretroviral treatment (ART) scale-up and access to TB care and treatment and is now being progressively applied to HCV and HBV testing and treatment scale-up [6, 43, 44]. While existing HIV viral load tests require laboratory infrastructure and highly trained technicians, POC devices can be battery operated, are easy to use as they require minimal hands on time (sample in-answer out), results are available in less than 2 h (shorter turnaround time) and reagents can be stored at ambient temperature. Most come with data transmission capability for automated surveillance, and the opportunity to link in quality assurance and supply chain software to avoid stock-outs [45]. POC qualitative assays are used widely for diagnosis of TB and quantitative assays for monitoring of ART treatment response by measuring HIV viral load. In Mozambique, it was demonstrated that POC HIV viral load testing is operationally feasible at the primary health care level [46]. Similarly, the roll-out of the POC TB assay has demonstrated that molecular diagnostics can produce rapid diagnosis and treatment initiation [47]. Such innovative technologies for HIV have been promoted as a means to reach the third 90 (undetectable viral load) of the UNAIDS/WHO 90–90-90 targets for HIV elimination, but also provide opportunities to test for other diseases [48–50].

Experience with use of POC virological diagnosis in viral hepatitis is largely confined to evaluation of HCV RNA, where the major potential value is to allow same day or next day confirmation of viraemic infection and so prompt initiation of treatment. The GeneXpert HCV viral load manufactured by Cepheid is currently the only WHO pre-qualified (PQ) molecular test for viral hepatitis and was included on the in vitro diagnostic (IVD) product list in April 2017. This complements the existing WHO PQ approval for the GeneXpert TB and HIV viral load instruments [51]. The major potential value of POC for HCV viral load is to allow potentially same day or next day confirmation of viraemic infection and so prompt initiation of treatment, but will also be important for confirmation of viral cure. It takes around 110 minutes for a result, and depending on the size of instrument (cartridge capacity) may run generally between 8 to 20 samples per day. The company has also developed a combo cartridge containing HIV-1 viral load (CE-IVD) and HBV viral load (for research use only).

At present there is only one HCV NAT qualitative product that it is close to commercialization. The Genedrive®, developed by the UK-based biotechnology company Epistem Ltd., is highly-portable and uses end-point PCR-based detection, and is expected to achieve European CE marking by the end of 2017. Sample preparation includes several manual steps that can hamper proper execution when performed by users with minimal laboratory training. Two HCV NAT qualitative products are also close to market approval. The GeneXpert Omni (Cepheid Inc.) is expected to be available in emerging markets in the third quarter of 2017 [52]. This device leverages on Cepheid existing cartridge technology with the objective of expanding access to clinical molecular diagnostic testing in decentralised locations. The company Molbio (Molbio Diagnostics Pvt. Ltd., India) aims to launch the Truenat HCV viral load assays in 2018. Truenat HCV is a chip based PCR test that runs on Molbio’s Truelab Uno Dx microPCR device, and uses the Trueprep Auto Sample station that automatically prepares the sample in approximately 20 min.

There is an evolving pipeline of other POC technologies for assessment of HCV viral load, and UNITAID will publish an updated landscape report on HCV diagnostics by the end of 2017 [14]. The biotechnology industry remains very active in developing NAT POC instruments with new functionalities. The small size of these devices makes them suitable for very small laboratories and/or mobile clinics, while features such as modularity and connectivity allow flexible uses and deployment options.

#### Use of HCVcAg as alternative to NAT

The HCV core antigen test was developed as an alternative to NAT, as access to and affordability of HCV confirmatory RNA NAT assays remains a challenge in resource-limited settings. The availability of a highly accurate immunoassay that can perform quantitative HCV cAg assays would be valuable in settings where NAT or near-NAT is not accessible, especially if it can reduce the cost of monitoring. HCVcAg assays may also be useful as a one-step screening test (as for NAT HCV RNA) in high prevalence populations or settings because HCVcAg appears earlier than HCV antibodies, and has a high specificity, so does not require any further confirmatory testing. Major advantages of HCVcAg assay in comparison to HCV RNA assays are cost per test, turnaround time and the random access to a platform that does not require specimen batching. However, the most widely used HCVcAg assay, a two-step chemiluminescent microparticle immunoassay (ARCHITECT HCV Ag), requires a floor-standing immunoassay analyser (ARCHITECT-i2000R, Abbott Diagnostics, Illinois, USA. A systematic review concluded that a well-performing HCVcAg test can have a similar diagnostic accuracy compared to NAT when the viral load is greater than 3000 IU/mL [13]. More recent data shows high sensitivity and specificity of HCVcAg when compared to HCV RNA for detection of viremia both before treatment and after treatment [53, 54]. The quantification of HCVcAg as a one-step approach in diagnosing chronic hepatitis C infection has also been evaluated in Cameroon and showed 95.7% sensitivity and 99.7% specificity, that was similar among those with HIV or HBV- coinfection [55]. Operational research studies are now needed to assess the relative utility, cost-effectiveness and challenges in use of HCVcAg compared to HCV RNA NAT in the context of national hepatitis programmes. The development of a highly sensitive POC platform that meets the requirements of the target product profile for a test of viraemia developed by FIND and WHO may also prove a more cost-effective option than NAT POC tests [56].

### Health system improvements

#### Integrated testing and decentralisation

Integration of viral hepatitis testing and care with other health services, and decentralization to lower level health facilities and task-shifting are all health system approaches that may improve the accessibility of hepatitis testing and linkage to care. The availability of simplified but highly effective DAA treatment regimens means that routine management of hepatitis C treatment can increasingly be provided outside of specialized centres and tertiary institutions, so the number of patients accessing viral hepatitis services also increased. The delivery of care and treatment of HCV infected PWID by primary health care providers was shown to be as safe and effective as that provided by specialists [57, 58]. Viral hepatitis services and particularly hepatitis B management in sub-Saharan Africa may also be integrated into the existing infrastructure of HIV programmes for testing, care and treatment.

Other recommended interventions to promote uptake of hepatitis testing and linkage to care include peer and lay health worker support in community-based settings, clinician reminders to offer testing, and testing as part of integrated services, alongside the use of different media strategies to promote awareness about hepatitis in the general population [6, 15, 59, 60].

#### Connectivity

Accurate and timely diagnostic test information is critical for the proper functioning of the diagnostic and treatment cascade including patient management, epidemiological surveillance, quality assurance, laboratory operations, and technical support. An accessible standardized database that enables integration with laboratory information systems, electronic health records, and health programme databases can allow diagnostics to meet these broad-ranging needs. Connectivity has become a default requirement for TB diagnostics and according to the WHO Framework of indicators and targets for laboratory strengthening under the End TB Strategy, all sites that use WHO-recommended rapid diagnostics should be transmitting results electronically to clinicians and to information management systems using data connectivity solutions no later than 2020 (Indicator 4) [61].

Connectivity solutions that link POC testing sites to a central laboratory also allow control programmes to track trends and assess the impact of special interventions. Many new molecular POC devices have data transmission capabilities via Wi-Fi or cellular networks, creating automated real-time surveillance systems [62]. For RDTs, readers or mobile phones can be used to standardize test interpretation, eliminating subjectivity. A microfluidic based serology test for HIV and syphilis antibody detection has been designed to be powered and read by a smart phone, thus automating data transmission to a central database [63]. Connectivity solutions can also improve the turn-around time of external quality assessment result submissions and allow corrective actions to be provided immediately [64]. It is essential that connectivity solutions address both the current and future needs of viral hepatitis programmes. These needs can include enhanced technical features and functions, integration with other systems or be financial in nature. It is also essential that innovative solutions are appropriately implemented to optimise utilization and resource use.

#### Supply chain and sample management

Supply chain management is one of the most challenging problems in health care delivery in the developing world [65]. As testing coverage increases with POC diagnostics reaching more remote areas, supply chain management will also become more decentralized, putting additional stresses on already fragile health systems suffering from a shortage of health care personnel and frequent stock outs. Innovative connectivity solutions can support the management of decentralized testing. The availability of connectivity solutions that can be installed on both high-throughput and POC devices may improve optimization of central supply chain and procurement mechanisms, by linking consumption data across multiple testing sites and for multiple diseases [64]. Similar solutions could be adopted to improve communication between peripheral clinics and central testing laboratories to optimize sample workflow [66]. For example, information could be transmitted to the testing laboratory as soon as a test is registered at the clinic. Realtime information on volumes expected would allow the testing laboratory to better plan their workflow.

## Conclusions and research agenda in viral hepatitis diagnostics

Innovations in testing and sampling approaches have the potential to increase access to testing and reduce the large burden of undiagnosed infection. These include the use of non-invasive sampling with oral fluid and potential self-testing among marginalized populations, although linkage to care will still represent a challenge. Multiplex platform molecular technologies will allow programmes to leverage investments in existing platforms for HIV or TB and increase access to testing for viral hepatitis. Additional areas to ensure successful adoption of new technologies include strengthening laboratory support systems, such as sample transport, quality management, and training.

There are well recognized research gaps in viral hepatitis diagnostics and many of these were highlighted throughout the WHO testing guidelines for chronic hepatitis B and C. These include both development of new innovative technologies, and high quality demonstration projects and implementation science to evaluate performance, impact and costs. In 2016 UNITAID awarded FIND a grant to address some of the challenges and operational questions relating to HCV diagnosis, working in collaboration with WHO and other partners. The key project goal is to advance progress towards the WHO elimination targets for HCV by 2030: 90% reduction in incidence; 65% reduction in mortality; and 80% of patients receiving treatment [5]. The key outcome is to support the updating of WHO normative guidelines on viral hepatitis testing as well as national policies that will support scale-up of HCV care and treatment. Specific activities and deliverables are summarized in Table 5 and include: 1) accelerating the development of new, simpler, quality-assured HCV tests and making them available in LMICs; 2) establishing innovative approaches for screening and treatment in HCV infected patients in 6 countries (Cameroon, Georgia, India, Myanmar, Viet Nam and Malaysia); 3) implementing a cost reduction strategy for diagnosis; and 4) providing the evidence to drive scale up, transition and sustainability.

**Table 5:** Areas of activity and key deliverables of FIND-UNITAID project

Areas of activity	Key deliverables
Enable platform polyvalence and HCV/HIV integration through supporting DBS protocol development, regulatory approval and policy change	- Evaluation of DBS sampling for centralized EIA and RNA assays - Use of existing HIV lab infrastructure for introducing HCV testing - Demonstrate service delivery advantages of one step sampling (EIA/ RNA) using DBS
Expand use of HCV core antigen assays	- Compare cAg against gold standard as test of cure using current lab-based technology - Evaluate feasibility, cost, cost-effectiveness and impact of cAg assay as one testing approach in high prevalence populations - Accelerate development of POC cAg devices
Enable decentralization of HCV services	- Integration of HCV/TB/HIV assays in decentralized settings - Demonstrate feasibility of HCV testing and treatment in different decentralized settings - Costing of different testing/ treatment pathways across countries and sites - Evaluate acceptability and cost-effectiveness of finger stick blood sample collection - Accelerate development of POC molecular devices
Increase access to quality-assured HCV RDTs	- Piloting quality-assured RDTs using capillary blood in high risk groups - Assess market and feasibility of HCV ST and/or combo test among different populations
